# Adequacy of early-stage breast cancer systemic adjuvant treatment to Saint Gallen-2013 statement: the MCC-Spain study

**DOI:** 10.1038/s41598-021-84825-2

**Published:** 2021-03-08

**Authors:** Inés Gómez-Acebo, Trinidad Dierssen-Sotos, Mónica Mirones, Beatriz Pérez-Gómez, Marcela Guevara, Pilar Amiano, Maria Sala, Antonio J. Molina, Jéssica Alonso-Molero, Victor Moreno, Claudia Suarez-Calleja, Ana Molina-Barceló, Juan Alguacil, Rafael Marcos-Gragera, María Fernández-Ortiz, Oscar Sanz-Guadarrama, Gemma Castaño-Vinyals, Leire Gil-Majuelo, Conchi Moreno-Iribas, Nuria Aragonés, Manolis Kogevinas, Marina Pollán, Javier Llorca

**Affiliations:** 1 CIBER Epidemiología y Salud Pública (CIBERESP), Madrid, Spain; 2 Universidad de Cantabria, Santander, Spain; 3 IDIVAL, Santander, Spain; 4 National Center for Epidemiology, Carlos III Institute of Health, Madrid, Spain; 5 Navarra Public Health Institute, Pamplona, Spain; 6 Navarra Institute for Health Research (IdiSNA), Pamplona, Spain; 7 Public Health Division of Gipuzkoa, Biodonostia Health Research Institute, Ministry of Health of the Basque Government, San Sebastian, Spain; 8 Department of Epidemiology and Evaluation, IMIM (Hospital del Mar Medical Research Institute), Barcelona, Spain; 9 Research Network on Health Services in Chronic Diseases (REDISSEC), Barcelona, Spain; 10 Grupo de Investigación en Interacción Gen-Ambiente-Salud (GIIGAS), Instituto de Biomedicina (IBIOMED), Universidad de León, León, Spain; 11 Oncology Data Analytics Program, Catalan Institute of Oncology (ICO), Hospitalet de Llobregat, Barcelona, Spain; 12 Colorectal Cancer Group, ONCOBELL Program, Bellvitge Biomedical Research Institute (IDIBELL), Hospitalet de Llobregat, Barcelona, Spain; 13 Department of Clinical Sciences, Faculty of Medicine, University of Barcelona, Barcelona, Spain; 14 Instituto de Investigación Sanitaria del Principado de Asturias-ISPA, Oviedo, Spain; 15 IUOPA, Universidad de Oviedo, Oviedo, Spain; 16 Cancer and Public Health Area, FISABIO-Public Health, Valencia, Spain; 17 Centro de Investigación en Recursos Naturales, Salud y Medio Ambiente (RENSMA), Universidad de Huelva, Huelva, Spain; 18 Epidemiology Unit and Girona Cancer Registry, Oncology Coordination Plan, Department of Health, Autonomous Government of Catalonia, Catalan Institute of Oncology, Girona, Spain; 19 Servicio de Cirugía General, Unidad de Mama, Complejo Asistencial Universitario de León, León, Spain; 20 ISGlobal, Barcelona, Spain; 21 IMIM (Hospital del Mar Medical Research Institute), Barcelona, Spain; 22 Universitat Pompeu Fabra (UPF), Barcelona, Spain; 23 Epidemiology Section, Public Health Division, Department of Health, Madrid, Spain; 24 Medicina Preventiva y Salud Pública, Facultad de Medicina, Avda. Herrera Oria s/n, 39011 Santander, Cantabria Spain

**Keywords:** Cancer, Health care, Medical research, Oncology, Risk factors

## Abstract

The St Gallen Conference endorsed in 2013 a series of recommendations on early breast cancer treatment. The main purpose of this article is to ascertain the clinical factors associated with St Gallen-2013 recommendations accomplishment. A cohort of 1152 breast cancer cases diagnosed with pathological stage < 3 in Spain between 2008 and 2013 was begun and then followed-up until 2017/2018. Data on patient and tumour characteristics were obtained from medical records, as well as their first line treatment. First line treatments were classified in three categories, according on whether they included the main St Gallen-2013 recommendations, more than those recommended or less than those recommended. Multinomial logistic regression models were carried out to identify factors associated with this classification and Weibull regression models were used to find out the relationship between this classification and survival. About half of the patients were treated according to St Gallen recommendations; 21% were treated over what was recommended and 33% received less treatment than recommended. Factors associated with treatment over the recommendations were stage II (relative risk ratio [RRR] = 4.2, 2.9–5.9), cancer positive to either progesterone (RRR = 8.1, 4.4–14.9) or oestrogen receptors (RRR = 5.7, 3.0–11.0). Instead, factors associated with lower probability of treatment over the recommendations were age (RRR = 0.7 each 10 years, 0.6–0.8), poor differentiation (RRR = 0.09, 0.04–0.19), HER2 positive (RRR = 0.46, 0.26–0.81) and triple negative cancer (RRR = 0.03, 0.01–0.11). Patients treated less than what was recommended in St Gallen had cancers in stage 0 (RRR = 21.6, 7.2–64.5), poorly differentiated (RRR = 1.9, 1.2–2.9), HER2 positive (RRR = 3.4, 2.4–4.9) and luminal B-like subtype (RRR = 3.6, 2.6–5.1). Women over 65 years old had a higher probability of being treated less than what was recommended if they had luminal B-like, HER2 or triple negative cancer. Treatment over St Gallen was associated with younger women and less severe cancers, while treatment under St Gallen was associated with older women, more severe cancers and cancers expressing HER2 receptors.

## Introduction

From 1995 on, many relevant advances in early breast cancer (BC) treatment have contributed to the improvement in patient survival. For instance, the emergence of anthracycline^1^ and taxane-based therapies^2^ and the identification of intrinsic subtypes^3^. Several guidelines for treating breast cancer patients have been published, although their recommendations differ only marginally^4^. In this regard, the 12th and 13th St Gallen International Breast Cancer Conference Expert Panel took place in 2011 and 2013, respectively; it endorsed a series of recommendations on early BC treatment^5,6^. We hereby briefly summarize St Gallen-2013 recommendations according to BC subtypes that are almost identical to those formulated in the previous meeting. Firstly, the Conference agreed in a clinico-pathological surrogate definition of intrinsic BC subtypes and secondly, the Conference stated systemic treatment recommendations for each subtype. In this regard, patients with luminal A-like BC should be treated with endocrine therapy, although cytotoxics may be added in the case of selected patients. Patients with luminal B-like (HER2 negative) BC should be treated with endocrine therapy and cytotoxics should be added for most of them. Patients with luminal B-like (HER2 positive) BC should receive cytotoxics, anti-HER2 and endocrine therapy; patients with HER2 positive (non-luminal) BC should receive cytotoxics and anti-HER2 and patients with triple negative (basal-like) BC should be treated with cytotoxics. It is noteworthy, that St Gallen panel was not unanimous in most of its decisions and it remarked that its recommendations were not just a blind guide; instead, “detailed decisions on treatment will, as always, involve clinical considerations of disease extent, host factors, patient preferences and social and economic constraints”^5^.

Several studies have shown adherence to guidelines for early stage BC diverged between countries^7^ and tumour characteristics; triple negative breast cancer^7,8^ and hormone receptors-negative HER2 positive^9^ being the subgroups with lower adherence. Older women are more likely to receive non-guideline adherent treatment leading to poorer survival rates^7,10,11^. Socio-economic status has been found to be associated to differences in guideline compliance in the US^12–14^ and to less extent in The Netherlands^15^.

The MCC-Spain breast cancer cohort recruited 1738 women with recent diagnosis of breast cancer in ten Spanish provinces between 2008 and 2013, which were subsequently followed-up until 2017 and 2018. In this paper, our objectives are: (1) to ascertain the clinical factors associated with St Gallen-2013 recommendations accomplishment, (2) to investigate whether there are differences by age and intrinsic BC subtype regarding St Gallen fulfilment, and (3) to examine the impact St Gallen non-fulfilment may have on survival with breast cancer. As most women in MCC-Spain were recruited before the 13th St Gallen Conference, St Gallen recommendations cannot be interpreted as a gold standard; therefore, our purpose is not to perform an audit but to identify patterns in actual clinical practice.

## Methods

### MCC-Spain BC cohort: setting and patients

MCC-Spain was born as a case–control study on colorectal, breast, prostate and gastric cancers and chronic lymphoid leukaemia^16^. Later on, the colorectal, breast and prostate cancer cases recruited in the case–control phase were incepted in three cancer-specific cohorts in order to ascertain clinical, genetic and epidemiological factors associated with prognosis^17^. From here on, we only refer to the breast cancer cohort.

This research was performed according to the standards required by the institutional research committees and the Declaration of Helsinki (last amendment, Fortaleza, 2013). The protocol of MCC-Spain was approved by each of the ethics committees of the participating institutions^16^. The specific study reported here was approved by the Ethical Committee of Clinical Research of Asturias, Barcelona, Cantabria, Girona, Gipuzkoa, Huelva, León, Madrid, Navarra and Valencia. Informed consent was obtained from all individual participants included in the study.

Women were included in the study cohort if they suffered an incident of pathologically confirmed breast cancer in the 2008–2013 period. After signing an informed consent, 1738 women were recruited in ten Spanish provinces (Asturias, Barcelona, Cantabria, Gipuzkoa, Girona, Huelva, León, Madrid, Navarra y Valencia) and were interviewed by trained personnel in order to gather demographic and epidemiological information^17^. Women with breast cancer in stages 0, I or II were included in this analysis (1214 patients).

### Tumour characteristics

Tumour characteristics at diagnosis were obtained from medical records. They included grade of differentiation (I: well, II: moderately, III: poorly differentiated), histological type (ductal, lobular, papillary, others), pathological stage according to TNM, presence or absence of oestrogen receptors, progesterone receptors and HER2 receptors, as well as other immunohistochemical properties when available (Ki-67, for instance)^17^. Intrinsic subtypes were determined according to St Gallen clinico-pathological surrogate definitions (Supplementary Table 1). A tumour was considered luminal A-like if it had positive oestrogen and progesterone receptors, HER2 negative and Ki-67 low. In this regard, tumours without Ki-67 determination were considered Ki-67 low for classification purposes if it was grade I or II, and Ki-67 high if it was grade III, as grade III is indicative of high proliferative activity, according to Curigliano et al.^18^. A tumour was classified as luminal B-like if (1) it had positive oestrogen receptors, HER2 negative and Ki-67 high or negative progesterone receptor or (2) positive oestrogen receptors and positive HER2. A cancer was considered HER2 positive (non-luminal) if it was oestrogen and progesterone receptors negative and HER2 receptors positive. Finally, tumours with oestrogen, progesterone and HER2 receptors negative were classified as triple negative.

### First-line treatment

Data on first-line treatment was obtained from medical records. They could include type of surgery (conservative/mastectomy), endocrine therapy, chemotherapy, anti-HER2 therapy or radiotherapy. Treatments other than surgery were classified as neoadjuvant, adjuvant or palliative, according to their purpose.

### Classification according to St Gallen-2013

First-line systemic treatments were classified into three groups (In-, Over- and Under St Gallen) according to the adherence to St Gallen-2013 recommendations as follows.

- In St Gallen: A treatment was considered “In St Gallen” if it consisted in exactly the main St Gallen recommendation for that patient (Supplementary Table 1). For instance, a woman with breast cancer oestrogen-receptor positive, progesterone-receptor positive and HER2 negative receiving just surgical treatment + endocrine therapy.
- Over St Gallen: A treatment was considered “Over St Gallen” if the woman received the main St Gallen recommendation for her cancer plus some additional therapy. For instance, a woman with breast cancer oestrogen-receptor positive, progesterone-receptor positive and HER2 negative receiving surgical treatment + endocrine therapy + anti-HER2 therapy.
- Under St Gallen: A treatment was considered “Under St Gallen” if the woman did not receive the main St Gallen recommendation for her cancer (in spite of whether she received additional treatments included in the main recommendation or not). For example, a woman with breast cancer oestrogen-receptor positive, progesterone-receptor positive and HER2 negative receiving surgical treatment + chemotherapy, but not endocrine therapy.

In order to analyse the reliability of the above indicated classification, two independent evaluators (MM and JA-M) applied it to a randomly selected 50-woman subsample, reaching a Cohen’s kappa = 0.76.

Follow-up for ascertaining the vital status was carried out in 2017 and 2018 by reviewing medical records, contacting women by phone and for women without contact with the hospital in the previous three months, by consulting the Spanish National Index of Death.

### Statistical analysis

The association between age or tumour characteristics and St Gallen fulfilment was analysed using multinomial logistic regression as the effect variable (St Gallen) has three categories; results—adjusted for age at diagnosis and hospital—are presented as relative risk ratios (RRR) with 95% confidence intervals. Overall survival is presented using Kaplan–Meier estimators. The association of St Gallen fulfilment and survival was analysed separately for each intrinsic subtype using Weibull regression adjusted for age at diagnosis, hospital, grade of differentiation and pathological stage. Weibull regression was considered adequate as the relationship between log[− log(survival probability)] and log(time of follow-up) was approximately linear. For this analysis, event was defined as death and patients were censored if they were alive at the end of follow-up. Weibull regression results are presented as hazard ratios (HR) with 95% confidence intervals. A complementary Weibull analysis was carried out where the event was distant recurrence or dead (the first that happened); patients alive and without distant recurrence at the end of follow-up were considered censored. All statistical analyses were performed with the Stata 16/SE software (Stata Co., College Station, Tx, US).

## Results

**Figure 1:**
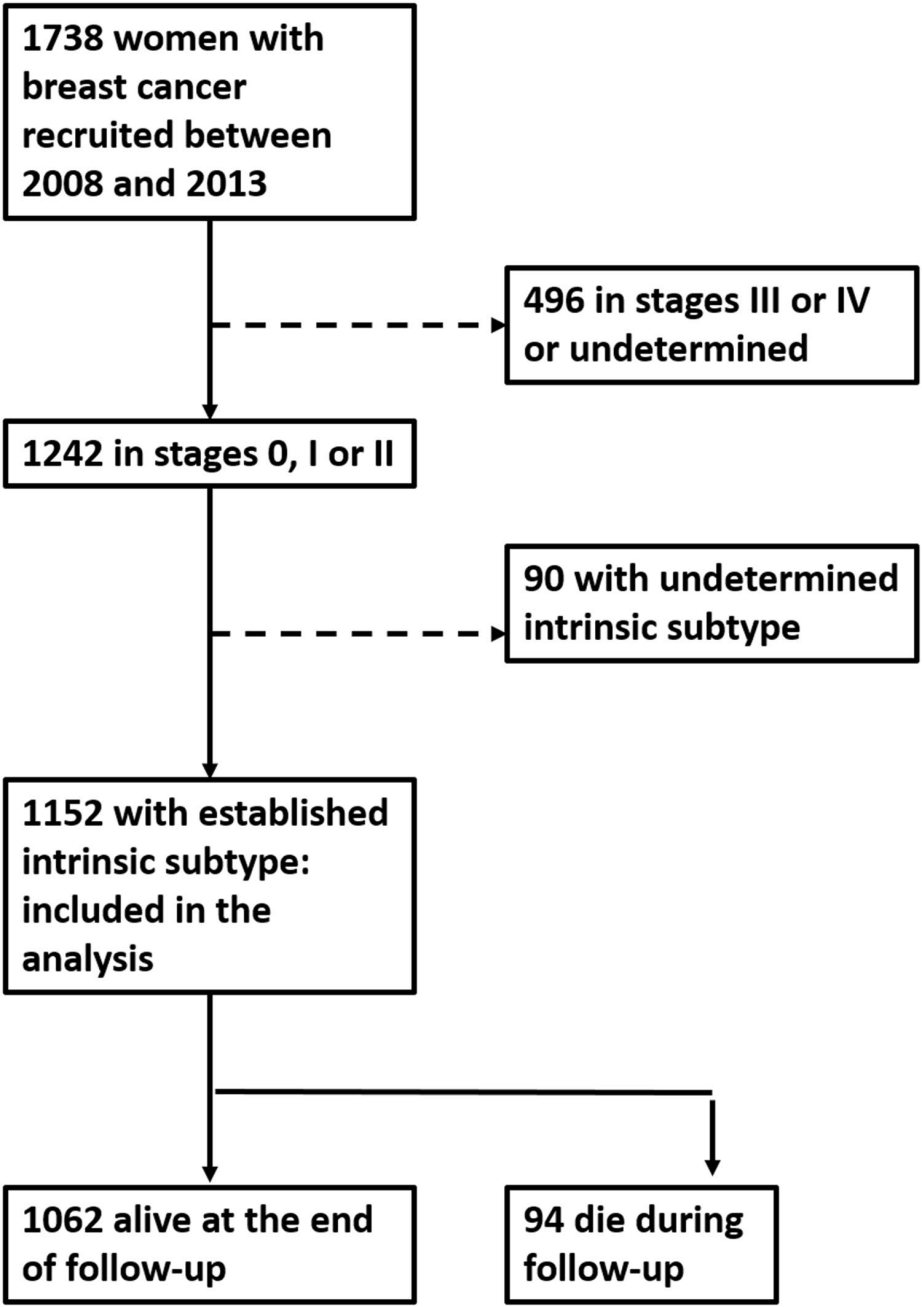
Flow chart of patients in this study. Solid arrows indicate patient selection. Dashed arrows indicate patient exclusion.

The study sample is described in Table 1 and the patient flow chart in Fig. 1. Out of 1242 breast cancers in stages 0, I or II, intrinsic subtype was established in 1152 cases; luminal A-like was the most frequent subtype (687 cases, 60%), 324 cases (28%) were luminal B-like, 52 (5%) were HER2 (non-luminal) and 89 (8%) were triple negative. The intrinsic breast cancer subtype could not be determined in 90 cases (7%). 56 were hormonal receptors positive, but results on HER2 receptor were not available. Therefore, they could be classified as luminal, but we could not ascertain if they were luminal A or luminal B. Eight cases were hormonal receptors negative, but—again—results on HER2 receptors were not available. Therefore, those cases were non-luminal, but we could not determine if they were HER-2 positive or triple negative. In 26 cases, we had no data on hormonal or HER2 receptors. Recurrence in the follow-up was found in 86 women (7.5%), 52 of them being distant. 94 (8.9%) women died in the follow-up.

**Table 1:** Description of the 1152 patients included in the sample.

Variable	Category	N (%)
Age (mean ± sd)		56.2 ± 12.2
Age	< 65	864 (75.0)
	≥ 65	288 (25.0)
Grade of differentiation	I (well differentiated)	260 (22.6)
	II (moderately differentiated)	367 (31.9)
	III (poorly differentiated)	222 (19.3)
	Missing	303 (26.3)
Histological type	Ductal	918 (79.7)
	Lobular	82 (7.1)
	Papilar	16 (1.4)
	Others	136 (11.8)
Pathological stage	0	36 (3.1)
	I	519 (45.1)
	II	597 (51.8)
Progesterone receptors	Negative	262 (22.7)
	Positive	887 (77.0)
	Missing	3 (0.3)
Oestrogen receptors	Negative	155 (13.5)
	Positive	996 (86.4)
	Missing	1 (0.1)
HER2 receptors	Negative	953 (82.7)
	Positive	199 (17.3)
Intrinsic subtype	Luminal A-like	687 (59.6)
	Luminal B-like	324 (28.1)
	HER2 positive (non-luminal)	52 (4.5)
	Triple negative (ductal)	89 (7.7)
Recurrence in the follow-up	No	1066 (92.5)
	Local	20 (1.7)
	Regional	9 (0.8)
	Distant	57 (5.0)
Vital status at the end of follow-up	Alive	1058 (91.1)
	Dead	94 (8.9)

Out of 1152 breast cancer cases with established intrinsic subtype, 523 (45.4%) were treated In St Gallen, 243 (21.1%) Over St Gallen and 386 (33.5) Under St Gallen. Over St Gallen was more frequent in patients with luminal A cancer (219 out of 687 cases, 31.9%) and Under St Gallen was more frequent in patients with luminal B cancer (173 out of 324 cases, 53.4%) (Table 2).

**Table 2:** Description of St Gallen fulfilment/non-fulfilment according to breast cancer intrinsic subtypes.

Intrinsic subtype	St Gallen fulfilment	Reason of non-fulfilment	n (%)^a^
Luminal A-like (n = 687)	In St Gallen		298 (43.4)
	Over St Gallen	Neoadjuvant + adjuvant chemotherapy	1 (0.1)
		Chemotherapy	220 (32.0)
	Under St Gallen	Lack of endocrine therapy	156 (22.7)
		Lack of chemotherapy	12 (1.7)
Luminal B-like (n = 324)	In St Gallen		139 (42.9)
	Over St Gallen	Neoadjuvant + adjuvant chemotherapy	12 (3.7)
	Under St Gallen	Lack of endocrine therapy	48 (14.8)
		Lack of chemotherapy	54 (16.7)
		Lack of anti-HER2 therapy	17 (5.3)
		Lack of chemotherapy and endocrine therapy	1 (0.5)
		No systemic treatment recorded	54 (16.7)
HER2 positive (non-luminal) (n = 52)	In St Gallen		17 (32.7)
	Over St Gallen	Neoadjuvant + adjuvant chemotherapy	7 (13.5)
	Under St Gallen	Lack of chemotherapy	1 (1.9)
		Lack of anti-HER2 therapy	13 (44.8)
		No systemic treatment recorded	14 (26.9)
Triple negative (ductal) (n = 89)	In St Gallen		69 (77.5)
	Over St Gallen	Anti-Her2 therapy	1 (1.1)
		Endocrine therapy	2 (2.3)
	Under St Gallen	Lack of chemotherapy	17 (19.1)
Total (n = 1152)	In St Gallen		523 (45.4)
	Over St Gallen		243 (21.1)
	Under St Gallen		386 (33.5)

^a^Percentages refer to each intrinsic subtype.

**Figure 2:**
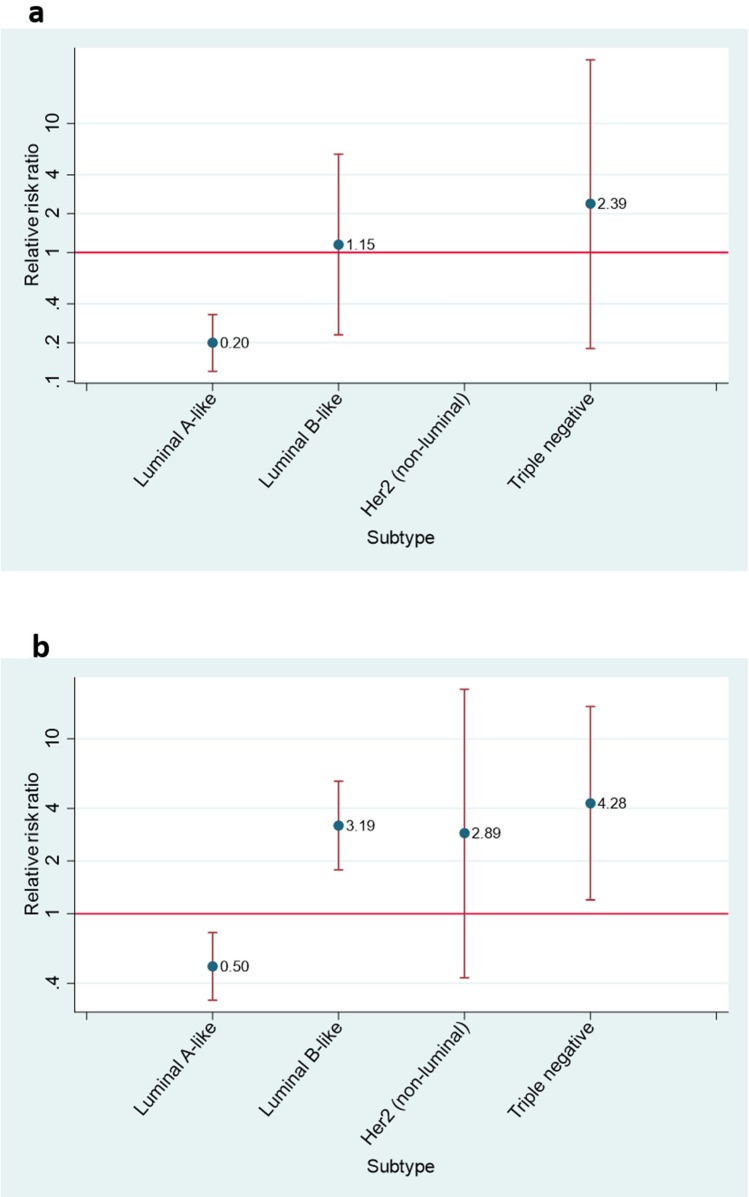
Relative risk ratios (RRR) for women over 65 years old compared with women under 65 years old of being treated Over (**a**) or Under (**b**) St Gallen. In (**a**), RRR > 1 indicates that women over 65 were more likely to be treated Over St Gallen than women under 65 years old, while in (**b**), RRR > 1 indicates that women over 65 were more likely to be treated Under St Gallen than women under 65. Results on HER2 + tumours treated Over St Gallen are not shown as analysis did not converge.

Table 3 provides the factors associated with Over St Gallen treatment. Women with breast cancer in stage II had more than four times the probability of being treated Over St Gallen (RRR = 4.15, 95% CI 2.90–5.94) compared to women with breast cancer in stage I. Cancers positive to either progesterone or oestrogen receptors also increased the likelihood of treatment Over St Gallen. Factors associated with lower probability of being treated Over St Gallen were age (RRR = 0.70, 95% CI 0.62–0.81 for each 10 years more), poorly differentiated cancers (RRR = 0.09, 95% CI 0.04–0.19), HER2 positive cancers (RRR = 0.46, 95% CI 0.26–0.81) and luminal B and triple negative subtypes (RRR = 0.07, 95% CI 0.04–0.13 and RRR = 0.03, 95% CI 0.01–0.11, respectively.) Regarding differences according to age by tumoral subtype, women over the age of 65 had a lower probability of being treated Over St Gallen if they had luminal A-like cancer than those under 65 (Fig. 2a).

**Table 3:** Tumour characteristics associated with “Over St Gallen” treatment.

Variable	Category	n Over St Gallen/n included	RRR (95% CI)	p
Age	(each 10 years)		0.70 (0.62–0.81)	< 0.001
Pathological stage	0	0/36	–	–
	I	56/519	1 (ref.)	–
	II	187/597	4.15 (2.90–5.94)	< 0.001
Grade of differentiation	I (well differentiated)	69/260	1 (ref.)	–
	II (moderately differentiated)	115/367	1.26 (0.84–1.89)	0.27
	III (poorly differentiated)	10/222	0.09 (0.04–0.19)	< 0.001
	Missing	49/303	1.03 (0.55–1.92)	0.94
Histological type	Ductal	200/918	1 (ref.)	–
	Lobular	20/82	1.72 (0.94–3.17)	0.08
	Papilar	1/16	0.31 (0.04–2.53)	0.28
	Others	22/136	0.87 (0.50–1.50)	0.61
Progesterone receptors	Negative	14/262	1 (ref.)	–
	Positive	229/887	8.12 (4.41–14.9)	< 0.001
	Missing	0/3	–	–
Oestrogen receptors	Negative	12/155	1 (ref.)	–
	Positive	231/996	5.71 (2.97–11.0)	< 0.001
	Missing	0/1	–	–
HER2 receptors	Negative	224/953	1 (ref.)	–
	Positive	19/199	0.46 (0.26–0.81)	0.007
Intrinsic subtype	Luminal A-like	221/687	1 (ref.)	–
	Luminal B-like	12/324	0.07 (0.04–0.13)	< 0.001
	HER2 positive (non-luminal)	7/52	0.30 (0.11–0.83)	0.02
	Triple negative (ductal)	3/89	0.03 (0.01–0.11)	< 0.001

Relative risk ratios (RRR) are adjusted for age at diagnosis and hospital of recruitment.

Under St Gallen treatment was more frequent in patients with pathological stage 0 (RRR = 21.6, 95% CI 7.221–64.5), poorly differentiated cancer (RRR = 1.88, 95% CI 1.21–2.93) or missing grade of differentiation (RRR = 3.72, 95% CI 2.21–6.29), cancers positive to HER2 receptors (RRR = 3.44, 95% CI 2.40–4.93) and luminal B and HER2 intrinsic subtypes (RRR = 3.63, 95% CI 2.59–5.09 and RRR = 4.38, 95% CI 2.23–8.60, respectively) (Table 4). Compared with women under 65, those 65 years or over had a higher probability of being treated Under St Gallen if they suffered a luminal B-like, Her2 (non-luminal) or triple negative cancer (Fig. 2b).

**Table 4:** Tumour characteristics associated with “Under St Gallen” treatment.

Variable	Category	n Under St Gallen/n included	RRR (95% CI)	p
Age	(each 10 years)		1.08 (0.06–1.21)	0.21
Pathological stage	0	32/36	21.6 (7.21–64.5)	< 0.001
	I	172/519	1 (ref.)	–
	II	182/597	1.34 (1.00–1.78)	0.05
Grade of differentiation	I (well differentiated)	61/260	1 (ref.)	–
	II (moderately differentiated)	97/367	1.38 (0.91–2.09)	0.13
	III (poorly differentiated)	84/222	1.88 (1.21–2.93)	0.005
	Missing	144/303	3.72 (2.21–6.29)	< 0.001
Histological type	Ductal	288/918	1 (ref.)	–
	Lobular	31/82	1.40 (0.82–2.41)	0.22
	Papilar	5/16	0.72 (0.23–2.25)	0.57
	Others	62/136	1.96 (1.28–2.99)	0.002
Progesterone receptors	Negative	110/262	1 (ref.)	–
	Positive	275/887	0.67 (0.49–0.94)	0.02
	Missing	1/3	0.67 (0.06–7.86)	0.75
Oestrogen receptors	Negative	51/155	1 (ref.)	–
	Positive	335/996	1.13 (0.76–1.67)	0.54
	Missing	0/1	–	–
HER2 receptors	Negative	272/953	1 (ref.)	–
	Positive	114/199	3.44 (2.40–4.93)	< 0.001
Intrinsic subtype	Luminal A-like	168/687	1 (ref.)	–
	Luminal B-like	173/324	3.63 (2.59–5.09)	< 0.001
	HER2 positive (non-luminal)	28/52	4.38 (2.23–8.60)	< 0.001
	Triple negative (ductal)	17/89	0.70 (0.39–1.28)	0.25

Relative risk ratios (RRR) are adjusted for age at diagnosis and hospital of recruitment.

**Figure 3:**
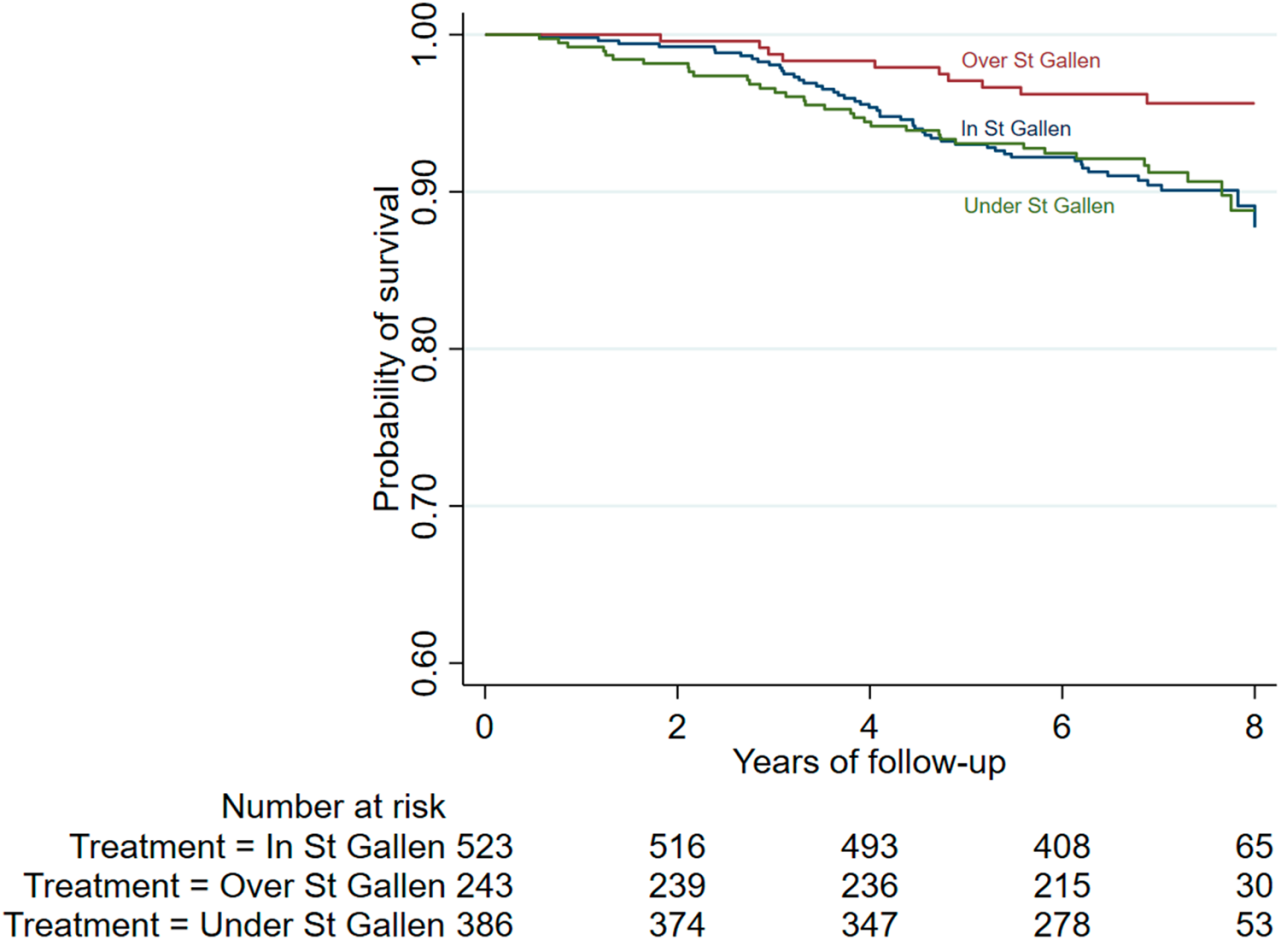
Crude Kaplan–Meier estimates of overall survival probability according to St Gallen fulfilment. Five-year survival probabilities were 97.1 for women treated over St Gallen, 93.1 for women treated under St Gallen and 93.0 for women treated in St Gallen.

The follow-up accounted for 7730 patient-years. 94 women died in the follow-up, making a linearized mortality rate of 1.22 per 100 patient-years (95% CI 0.98–1.49). Crude 5-year overall survival was 94.2%; survival at 5 years was approximately equal for patients treated In St Gallen and Under St Gallen and about 4% higher for patients Over St Gallen (Table 5, Fig. 3). After adjusting for age, hospital, grading and stage at diagnosis, Under St Gallen treatment was associated with a higher probability of dying in women with triple negative breast cancer (HR = 4.65, 95% CI 0.87–24.8), but not in other breast cancer subtypes (Suppl. Table 2). Results from Weibull regression using the combined event distant recurrence or dead (Suppl. Table 3) were similar to those founded for overall survival (Suppl. Table 2).

**Table 5:** Crude Kaplan–Meier estimated survival probabilities according to St Gallen adherence and age at diagnosis.

	St Gallen adherence	5-year overall survival probability^a^	5-year survival probability without distant recurrence^b^
Whole sample	In St Gallen	93.0 (90.4–94.9)	91.5 (88.7–93.6)
	Over St Gallen	97.1 (94.0–98.6)	96.7 (93.4–98.3)
	Under St Gallen	93.1 (90.0–95.2)	92.6 (89.4–94.8)
	Total	94.2 (92.7–95.4)	92.9 (91.3–94.3)
Age < 65	In St Gallen	94.9 (92.1–96.7)	93.3 (90.3–95.4)
	Over St Gallen	97.6 (94.3–99.0)	97.1 (93.7–98.7)
	Under St Gallen	96.3 (93.2–98.0)	95.9 (92.7–97.7)
	Total	96.3 (94.9–97.3)	95.1 (93.4–96.3)
Age ≥ 65	In St Gallen	88.1 (81.5–92.4)	86.7 (79.9–91.3)
	Over St Gallen	93.6 (76.6–98.4)	93.6 (76.6–98.4)
	Under St Gallen	84.9 (76.6–90.5)	84.0 (75.6–89.8)
	Total	86.7 (82.0–90.3)	86.4 (81.8–86.4)

^a^Event: dead. Censored: patients alive at the end of follow-up.

^b^Event: dead or distant recurrence. Censored: patients alive and without distant recurrence at the end of follow-up.

## Discussion

According to our results, systemic treatment of early breast cancer fully accomplished St Gallen-2013 recommendations in about 50% patients; two out of nine were treated Over St Gallen and three in ten were treated Under St Gallen. This variability was related to both age and tumour characteristics. Regarding age, older women tended to have less likelihood of being treated Over St Gallen (30% lower probability each ten years) and more likelihood, although non-statistically significant- to be treated Under St Gallen (8% higher probability each ten years). Altogether, more severe tumours (stage II, poorly differentiated, hormone receptors negative, basal-like) were less likely to be treated Over St Gallen. Most severity factors, except triple negative tumour, were associated with higher probability of being treated Under St Gallen.

Interpreting these results is not straightforward. First of all, St Gallen recommendations were not just a “must”. Instead, they should have been individualized in the light of both patient and clinical information. In this regard, further St Gallen International Breast Cancer Conferences have focused on a practical approach of therapies to individual patients^19^ as well as identifying patients that could benefit from escalating or de-escalating treatments^18^. Our results cannot be interpreted as an audit of accomplishing clinical guides as patients in our cohort were recruited from 2008 to 2013 (i.e., before St Gallen recommendations were stated); instead, our comparison of actual treatments with St Gallen is a portrayal on the way breast cancer patients were treated as compared to the state-of-the-art about the same time.

Apart from St Gallen recommendations, several organizations have delivered their own guidelines on breast cancer treatment^20,21^, although they usually differ only marginally^4^. A main factor related with deviations from guidelines is woman’s age. Several studies have described that older women are less likely to receive or be offered standard treatment^10,11,22–24^, leading to be given adjuvant chemotherapy less frequently^25,26^. Regarding endocrine therapy, between-countries large variation has been observed in women over 70 years old, without variation in relative survival, which suggests possible overtreatment^27^; women under 50 have been found less adherent to endocrine therapy^28^. According to our results, treatment in Spanish women with breast cancer differed between those over and under 65 years of age. The latter being less prone to be treated Over St Gallen if suffering luminal A-like and more prone to be treated Under St Gallen if suffering any other intrinsic breast cancer subtype (luminal B-like, Her2 non-luminal or triple negative), meaning that in all subtypes, women over 65 received less treatment on average. There are several issues regarding recommendations for treating early breast cancer in women over 65 or 70; firstly, breast cancers in older women tend to be less aggressive^29^. Secondly, older women are usually under-represented in clinical trials^30,31^, which makes it more difficult to establish standards for treating these patients^32^. Thirdly, Spanish women aged 65 and 70 have life expectancy of 23.0 and 18.7 years, respectively^33^; therefore, short expectancy of life cannot be argued for supporting undertreatment.

Women with luminal A-like tumours were more probably treated Over St Gallen than women with any other intrinsic subtype. In this regard, the main Over St Gallen treatment in women with luminal A-like tumours was chemotherapy. Whether hormone-positive, HER2-negative and node-negative patients would benefit from chemotherapy remains controversial; a 21-gene score (Oncotype DX, Genomic Health, Redwood City, CA) has been proven to have predictive value for recurrence^34,35^ and has been endorsed by several scientific societies^20,21^. TAILORx trial has shown that women scoring Oncotype DX ≤ 25 can receive hormone therapy alone, while women scoring > 25 should benefit from adjuvant chemotherapy^36,37^. By the time our patients were recruited, genetic testing was not of general use. However, it has been recently shown that combining information from age, tumour size, grading, progesterone receptors and histological type can establish risk of recurrence as from Oncotype DX^38^. When applying Orucevic et al. model to our patients, only one in ten women receiving chemotherapy against luminal A-like tumour had a probability higher than 20% of having a high risk of recurrence, which suggests most of them had been over treated (results not shown).

Our results could imply some clinical considerations. First of all, St Gallen recommendations, as well as guidelines issued by other organizations^20,21^, could inform about treatment of patients with BC, although clinicians should take decisions on an individual basis. In this regard, the trend we describe towards less aggressive treatment in older women is noteworthy. Such a conservative decision could not be justified by general considerations (e.g., expectancy of life in older women), but on specific individual grounds (e.g., comorbidities or other factors limiting patient’s benefit). Secondly, the idea of BC being a homogenous disease requiring homogenous treatment is largely outdated. However, we lay out the fact that less aggressive BC tend to be treated over the standard recommendations while those that are more aggressive are treated under the recommendations, which makes treatment of biologically different BC as if they were alike. The clinical consequences of it would require further research.

Our study has some limitations. Firstly, our classification on St Gallen recommendation accomplishment is somewhat subjective; we have found the between-raters reliability to be high, but there is still room for misclassification. Secondly, less severe cancers usually require less treatment and so, are more likely to be Over St Gallen, while more severe cancers require more treatment and are more likely to be Under St Gallen. Thirdly, our follow-up is still short as 5-year survival in early BC is around 90%. Fourthly, comorbidities were not recorded, which may affect whether clinical guidelines are closely followed or not. In fifth place, our number of patients—although high for general analysis—was not enough to study interactions or to analyse the effect of St Gallen unfulfillment in depth. In this regard, several results of our survival analysis are based on small figures (Suppl. Table 2), which makes them little reliable. Our study also has some strengths. Firstly, information on more than one thousand patients was obtained in a standardized way without acknowledgment of this paper’s hypothesis; therefore, misclassification on clinical characteristics or first-line treatment could only introduce a non-differential bias. Secondly, follow-up was performed prospectively, which guarantees high quality follow-up data.

In conclusion, about 50% women with early BC were treated according to St Gallen recommendations. Treatment Over St Gallen was associated with younger women and less severe cancers (luminal A-like, well-differentiated, stage I) and treatment Under St Gallen was associated with older women, more severe cancers and cancers expressing HER2 receptors. No differences in overall survival were observed between the Under St Gallen group compared to the adherent group, which implies that there were no great deviations from “standard” treatment in our context. Finally, the improvement in survival observed in the Over St Gallen group supports the decision taken by the medical team treating these patients.

## Supplementary Information

Supplementary Information.
