# Supplementary material for: Adequacy of early-stage breast cancer systemic adjuvant treatment to Saint Gallen-2013 statement: the MCC-Spain study
Source: Sci Rep. 2021 Mar 8;11:5375. doi: 10.1038/s41598-021-84825-2 (PMC7970883; doi:10.1038/s41598-021-84825-2)
Supplement: Supplementary file 1 — Supplementary Information. [file 41598_2021_84825_MOESM1_ESM.docx]

**TITLE PAGE**

**Full title:** Adequacy of early-stage breast cancer systemic adjuvant treatment to Saint Gallen-2013 statement: the MCC-Spain study

**Short title:** Adequacy of early-stage breast cancer

**Authors:**

Inés Gómez-Acebo^1,2,3^, Trinidad Dierssen-Sotos^1,2,3^, Mónica Mirones^2^, Beatriz Pérez-Gómez^1,4^, Marcela Guevara^1,5,6^, Pilar Amiano^1,7^, Maria Sala^8,9^, Antonio J Molina^10^, Jéssica Alonso-Molero^2,3^, Victor Moreno^1,11,12,13^, Claudia Suarez-Calleja^1,14,15^, Ana Molina-Barceló^16^, Juan Alguacil^1,17^, Rafael Marcos-Gragera^1,18^, María Fernández-Ortiz^3^, Oscar Sanz-Guadarrama^19^, Gemma Castaño-Vinyals^1,20,21,22^, Leire Gil-Majuelo^7^, Conchi Moreno-Iribas^5,6^, Nuria Aragonés^1,23^, Manolis Kogevinas^1,20,21,22^, Marina Pollán^1,4^, Javier Llorca^1,2,3^

**Affiliations:**

1: CIBER Epidemiología y Salud Pública (CIBERESP), Madrid, Spain

2: Universidad de Cantabria, Santander, Spain

3: IDIVAL, Santander, Spain

4: National Center for Epidemiology, Carlos III Institute of Health, Madrid, Spain

5: Navarra Public Health Institute, Pamplona, Spain

6: Navarra Institute for Health Research (IdiSNA), Pamplona, Spain

7: Public Health Division of Gipuzkoa, Biodonostia Health Research Institute, Ministry of Health of the Basque Government, San Sebastian, Spain

8: Department of Epidemiology and Evaluation, IMIM (Hospital del Mar Medical Research Institute), Barcelona, Spain.

9: Research Network on Health Services in Chronic Diseases (REDISSEC), Barcelona, Spain

10: Grupo de Investigación en Interacción Gen-Ambiente-Salud (GIIGAS). Instituto de Biomedicina (IBIOMED), Universidad de León, León, Spain

11: Oncology Data Analytics Program, Catalan Institute of Oncology (ICO). Hospitalet de Llobregat, Barcelona, Spain

12: Colorectal Cancer Group, ONCOBELL Program, Bellvitge Biomedical Research Institute (IDIBELL). Hospitalet de Llobregat, Barcelona, Spain

13: Department of Clinical Sciences, Faculty of Medicine, University of Barcelona, Barcelona, Spain

14: Instituto de Investigación Sanitaria del Principado de Asturias – ISPA, Oviedo, Spain

15: IUOPA, Universidad de Oviedo, Oviedo, Spain

16: Cancer and Public Health Area, FISABIO – Public Health, Valencia, Spain

17: Centro de Investigación en Recursos Naturales, Salud y Medio Ambiente (RENSMA), Universidad de Huelva, Huelva, Spain

18: Epidemiology Unit and Girona Cancer Registry. Oncology Coordination Plan, Department of Health, Autonomous Government of Catalonia, Catalan Institute of Oncology, Girona, Spain

19: Servicio de Cirugía General, Unidad de Mama, Complejo Asistencial Universitario de León, León, Spain

20: ISGlobal, Barcelona, Spain

21: IMIM (Hospital del Mar Medical Research Institute), Barcelona, Spain

22: Universitat Pompeu Fabra (UPF), Barcelona, Spain

23: Epidemiology Section, Public Health Division, Department of Health, Madrid, Spain

**Corresponding author:**

Ines Gómez-Acebo

Medicina Preventiva y Salud Pública

Facultad de Medicina

Avda. Herrera Oria s/n

39011 Santander

Cantabria

Phone #34-942201993

Fax #34-942201903

E-mail: ines.gomez@unican.es

Supplementary Table 1. Main St Gallen – 2013 recommendations (modified from Goldhirsch et al, 2013, Tables 2 and 3).

| **Intrinsic subtype** | **Clinico-pathologic surrogate definition** | **Type of therapy** |
| --- | --- | --- |
| **Luminal A** | **“Luminal A-like”**   - ER and PgR positive - HER2 negative - Ki-67 “low” - Recurrence risk “low” based on multi-gene-expression assay (if available) | Endocrine therapy (often used alone).  Relative indications for the addition of cytotoxics included:  high 21-gene RS, if available  70-gene high risk status, if available  grade 3 disease  involvement of four or more lymph nodes |
| **Luminal B** | **“Luminal B-like (HER2 negative)”**   - ER positive - HER2 negative - and at least one of:   - Ki-67 “high”   - PgR negative or “low”   - Recurrence risk “high” based on multi-gene-expression assay (if available) | Endocrine therapy for all patients, cytotoxic therapy for most |
|  | **“Luminal B-like (HER2 positive)”**   - ER positive - HER2 over-expressed or amplified - Any Ki-67 - Any PgR | Cytotoxics + anti-HER2 + endocrine therapy |
| **Erb-B2 overexpression** | **“HER2 positive (non-luminal)”**   - HER2 over-expressed or amplified - ER and PgR absent | Cytotoxics + anti-HER2 |
| **Basal-like** | **“Triple negative (ductal)”**   - ER and PgR absent - HER2 negative | Cytotoxics |

Supplementary Table 2. Overall survivorship according to breast cancer subtype, St Gallen fulfilment and age at diagnosis

| **Breast cancer subtype** | **Variable** | **Category** | **n dead/n included** | **Hazard ratio (95% CI)** | **p** |
| --- | --- | --- | --- | --- | --- |
| **Luminal A-like** | **St Gallen fulfilment^i^** | **In St Gallen** | 23/298 | 1 (ref.) | - |
|  |  | **Over St Gallen** | 8/221 | 0.63 (0.25 – 1.62) | 0.34 |
|  |  | **Under St Gallen** | 11/168 | 0.61 (0.27 – 1.38) | 0.23 |
|  | **Age^ii^** | **<65 y.** | 16/509 | 1 (ref.) | - |
|  |  | **>65 y.** | 26/178 | 5.92 (3.03 – 11.6) | <0.001 |
| **Luminal B-like** | **St Gallen fulfilment^i^** | **In St Gallen** | 11/139 | 1 (ref.) | - |
|  |  | **Over St Gallen** | 1/12 | 0.86 (0.10 – 7.70) | 0.89 |
|  |  | **Under St Gallen** | 13/173 | 0.70 (0.26 – 1.90) | 0.48 |
|  | **Age^ii^** | **<65 y.** | 10/245 | 1 (ref.) | - |
|  |  | **>65 y.** | 15/79 | 5.71 (2.28 – 14.2) | <0.001 |
| **Her2-like (non-luminal)** | **St Gallen fulfilment^i^** | **In St Gallen** | 4/17 | 1 (ref.) | - |
|  |  | **Over St Gallen** | 0/7 | Non estimable | - |
|  |  | **Under St Gallen** | 3/28 | 0.09 (0.00 – 3.41) | 0.20 |
|  | **Age^ii^** | **<65 y.** | 5/42 | 1 (ref.) | - |
|  |  | **>65 y.** | 2/10 | 7.90 (0.47 – 133.1) | 0.15 |
| **Basal-like (triple negative)** | **St Gallen fulfilment^i^** | **In St Gallen** | 12/69 | 1 (ref.) | - |
|  |  | **Over St Gallen** | 1/3 | 0.34 (0.02 – 6.20) | 0.47 |
|  |  | **Under St Gallen** | 7/17 | 4.65 (0.87 – 24.8) | 0.07 |
|  | **Age^ii^** | **<65 y.** | 13/68 | 1 (ref.) | - |
|  |  | **>65 y.** | 7/21 | 2.70 (0.77 – 9.45) | 0.12 |

i: Hazard ratios adjusted for age, hospital, grading and stage at diagnosis. ii: Hazard ratios adjusted for St Gallen fulfilment, hospital, grading and stage at diagnosis

Supplementary Table 3. Survivorship without distant recurrence according to breast cancer subtype, St Gallen fulfilment and age at diagnosis

| **Breast cancer subtype** | **Variable** | **Category** | **n dead or distant recurrence/n included** | **Hazard ratio (95% CI)** | **p** |
| --- | --- | --- | --- | --- | --- |
| **Luminal A-like** | **St Gallen fulfilment^i^** | **In St Gallen** | 26/298 | 1 (ref.) | - |
|  |  | **Over St Gallen** | 10/221 | 0.57 (0.25-1.33) | 0.20 |
|  |  | **Under St Gallen** | 16/168 | 1.03 (0.49-2.16) | 0.93 |
|  | **Age^ii^** | **<65 y.** | 16/509 | 1 (ref.) | - |
|  |  | **>65 y.** | 26/178 | 3.90 (2.17-6.97) | <0.001 |
| **Luminal B-like** | **St Gallen fulfilment^i^** | **In St Gallen** | 13/139 | 1 (ref.) | - |
|  |  | **Over St Gallen** | 1/12 | 0.76 (0.09-6.34) | 0.80 |
|  |  | **Under St Gallen** | 116/173 | 0.77 (0.32-1.87) | 0.57 |
|  | **Age^ii^** | **<65 y.** | 10/245 | 1 (ref.) | - |
|  |  | **>65 y.** | 15/79 | 3.44 (1.53-7.74) | 0.003 |
| **Her2-like (non-luminal)** | **St Gallen fulfilment^i^** | **In St Gallen** | 4/17 | 1 (ref.) | - |
|  |  | **Over St Gallen** | 0/7 | Non-estimable | - |
|  |  | **Under St Gallen** | 4/28 | 0.32 (0.03-3.59) | 0.36 |
|  | **Age^ii^** | **<65 y.** | 5/42 | 1 (ref.) | - |
|  |  | **>65 y.** | 2/10 | 2.99 (0.31-28.7) | 0.34 |
| **Basal-like (triple negative)** | **St Gallen fulfilment^i^** | **In St Gallen** | 12/69 | 1 (ref.) | - |
|  |  | **Over St Gallen** | 1/3 | 0.45 (0.03-6.81) | 0.57 |
|  |  | **Under St Gallen** | 7/17 | 5.67 (1.07-30.0) | 0.04 |
|  | **Age^ii^** | **<65 y.** | 13/68 | 1 (ref.) | - |
|  |  | **>65 y.** | 7/21 | 2.37 (0.68-8.31) | 0.18 |

i: Hazard ratios adjusted for age, hospital, grading and stage at diagnosis. ii: Hazard ratios adjusted for St Gallen fulfilment, hospital, grading and stage at diagnosis
